# Feasibility of exercise training to address corticosteroid-induced changes in adolescents aged 10+ with lymphoma or leukemia during the first eight weeks of treatment: a pilot study

**DOI:** 10.3389/fped.2026.1819451

**Published:** 2026-10-07

**Authors:** Vera Blümer, Sarah Erhard, Nicola Schanda, Aleksandar Tomaskovic, Mirjam Dieckelmann

**Affiliations:** Goethe University Frankfurt, Department of Pediatrics, Frankfurt (Main), Germany

**Keywords:** body composition, child, exercise therapy, leukemia, lymphoma, pediatric oncology, physical functioning, pilot study

## Abstract

**Introduction:**

Children and adolescents with hematological malignancies are at risk of adverse side effects from high-dose corticosteroid treatment during induction therapy. In this context, exercise and nutrition interventions are promising, physiologically grounded approaches but their feasibility and practical delivery during early intensive therapy remain unclear. Therefore, this prospective pilot randomized controlled trial was designed to evaluate the feasibility and preliminary effects of an exercise intervention and nutrition consultation in newly diagnosed patients.

**Methods:**

Participants with leukemia or lymphoma, aged 10–17 years, were allocated to an 8-week exercise or sham control intervention (stratified randomization, 1:1 ratio, parallel groups) plus one-time nutrition counseling. Feasibility outcomes included recruitment, retention, adherence, safety, completion of assessments, and subjective feedback from patients and personnel involved in the study. Furthermore, repeated measures of physical performance, functional capacity, metabolic health, and body composition were used to explore baseline status and changes over time, after four and eight weeks of therapy.

**Results:**

Six participants, two of which were allocated to the intervention group, completed the trial. Overall rates of consent (67%), training adherence (51%) and complete measures (70%) did not attain prespecified criteria (≥80%). Despite considerable variability, pilot data indicated reduced physical capacity and functioning inferior to healthy norms at baseline and over the course of the intervention including hand grip strength (Δ*Z* ± SD = −1.5 ± 1.4), cardiorespiratory fitness (steep ramp cycle ergometry peak power; Δ*Z* ± SD = −3.8 ± 1.2), and lower extremity muscle strength (5-times sit-to-stand test; Δ*Z* ± SD = 2.3 ± 2.0), while having mostly normal quadriceps muscle and abdominal fat thickness and erratic unfavorable metabolic states. No formal exploration of intervention effects was performed.

**Discussion:**

Limited feasibility opposed continuation to a definite trial. Respective challenges observed may reflect particularities of early treatment, thus, substantial changes are advised for future protocols (e.g., towards a more adaptive trial design). Besides their limited generalizability and variability, pilot data suggests that adolescents undergoing induction therapy can experience intermittent deconditioning, while other outcomes were inconclusive. Thus, future exercise interventions should account for various objections to exercise participation, heterogeneous medical conditions, and potentially impaired physical capacity and functioning, especially during early therapy.

**Clinical Trial Registration:**

https://drks.de/search/de/trial/DRKS00032259, identifier DRKS00032259

## 1 Introduction

Rapid declines in physical capacity, including losses in muscle strength and reductions in functional mobility, are common during intensive therapy for childhood hematologic malignancies such as leukemias and lymphomas (1, 2). During early therapy (i.e., induction), high-dose corticosteroids are indispensable components due to their immunosuppressive and antileukemic effects (4). However, their potent pharmacological action is accompanied by a broad spectrum of metabolic, neuromuscular, skeletal, and behavioral toxicities, including hyperglycemia and insulin resistance (5, 6), accelerated protein catabolism and muscle atrophy (7, 8), shifts in body composition towards increased adiposity (9), as well as impaired bone turnover and osteonecrosis (10, 11). Susceptibility to corticosteroid-induced toxicities varies markedly across patients but pre-existing obesity, reduced cardiorespiratory or muscular fitness at diagnosis, high-risk disease, or older age within the pediatric range (≥10 years) have been found to increase the risk for pronounced metabolic and functional deterioration (3, 12–14).

With survival rates continuing to rise, counteracting such acute toxicities and mitigating long-term treatment complications has become a central priority in pediatric oncology (15). Even transient impairments can be clinically relevant since early deficits in metabolic regulation and physical capacity increase the likelihood of persistent cardiometabolic and musculoskeletal late effects in survivorship (16, 17). In this context, exercise and nutrition interventions represent promising, physiologically grounded approaches to counteract corticosteroid-induced alterations. In healthy children and adolescents, aerobic exercise acutely improves insulin sensitivity and increased skeletal muscle glucose uptake (18, 19). Similar benefits following resistance exercise seen in adults have been postulated for children and adolescents (20). In addition, nutritional interventions (e.g., counselling) aim to prevent dietary inadequacies (21) and ensure adequate protein intake for muscle protein synthesis (22, 23). To date, however, clinical management of corticosteroid-induced side effects remains largely reactive, as routine monitoring only enables detection of clinically manifest abnormalities but does not help to alleviate their emergence.

While the physiological potential of exercise and nutrition is well-established, there is limited evidence on whether it is feasible to introduce an exercise intervention and nutrition counselling immediately following diagnosis (24–26). Also, practical considerations of repeated assessments and intervention delivery that are inherent to high-quality research remain largely unknown. For instance, implementation may be limited by fluctuating symptom burden [e.g., fatigue, nausea; (27)], inconsistent treatment schedules, hospitalization-related constraints, psychological distress, and/or physical changes. Correspondingly, exercise therapists are challenged to find the optimal dose of exercise by carefully balancing these circumstances, patients' needs (28), potential stressors and risks (26), as well as assumed physiological (29–31) and psychological benefits (32). On that note, a pilot randomized controlled trial (pilot RCT) is essential to determine whether study procedures, outcome measures, and intervention delivery are feasible before initiating a definite trial powered for efficacy. Among others, key aspects of trial feasibility include the availability of and acceptance across eligible participants, the retention and adherence of those enrolled as well as protocol fidelity without compromising patient safety (33).

Therefore, this pilot RCT was designed to evaluate the feasibility and preliminary effects of a combined exercise and nutrition intervention delivered during the first eight weeks of treatment in adolescents with leukemia and lymphoma. Specifically, objectives were to assess whether an RCT design is feasible to be conducted during early treatment in terms of successful recruitment, retention, adherence, safety, and completion of outcome assessments, as well as for exploring changes over time and preliminary intervention-related effects in physical performance, functional capacity, metabolic health, and body composition.

## 2 Methods

### 2.1 Study design

This study was originally designed as single center, assessor-blinded, two-armed pilot RCT with parallel group allocation (1:1 ratio; exercise intervention plus nutrition vs. sham control plus nutrition). The study took place at the Department of Pediatrics at the University Medical Center Frankfurt am Main, Germany. The study was registered at the German Clinical Trials Register (DRKS; identification number DRKS00032259) and received ethics approval by the Ethics Committee of the Department of Medicine (University Hospital of Goethe University, Nr. E75/23). Written informed consent was obtained from all participants and/or their legal guardians prior to enrollment. An overview of the study protocol, including assessment time-points and intervention is available as Supplementary Material.

Prior to trial initiation, a participatory co-creation phase (36) was conducted to support the development of the exercise training intervention (see Supplementary Material). Following the clinical diagnosis of leukemia or lymphoma, eligible patients were screened within the first days after diagnosis and invited to participate in the study. After providing informed consent, participants were enrolled and randomly allocated (1:1) to either the multimodal exercise intervention group or a sham exercise control group. In both groups, one initial nutrition counselling was conducted to ensure standardized knowledge about nutrition during cancer treatment.

Baseline assessments were performed at study entry (t0), followed by repeated assessments at defined time points during the first eight weeks of therapy, such as within week one (t1), week four (t2), and week eight (t3). The intervention period comprised the initial eight weeks, after which participants completed usual care as post-intervention and one subsequent follow-up assessment (t4). After the trial commenced, no amendments were made to the protocol.

### 2.2 Participants

#### 2.2.1 Eligibility criteria

Children and adolescents were eligible to participate if they were between 10 and 17 years of age, newly diagnosed with leukemia or lymphoma, and scheduled to receive high-dose corticosteroid therapy as part of standard treatment protocols (37) used at the Department of Pediatric Hematology and Oncology at the University Medical Center Frankfurt. Eligible diagnostic groups included acute lymphoblastic leukemia (ALL), Non-Hodgkin lymphoma (NHL), and Hodgkin lymphoma (HL), those of which involve high-dose corticosteroid administration from the day of diagnosis.

Patients were excluded if cognitive impairments, insufficient German or English language proficiency, or a physician-determined contraindication to exercise participation at diagnosis prevented safe or reliable involvement in the study. During the intervention period, temporary medical conditions that could compromise exercise safety resulted in temporary suspension from participation in the exercise training sessions but did not study withdrawal (contraindications listed under subheading protocol adherence).

Study participation was discontinued if patients withdrew consent, were transferred to intensive care after enrollment and remained there for two weeks, changed treatment sites, or lacked medical clearance for supervised exercise therapy for two consecutive weeks.

#### 2.2.2 Recruitment

Participants were recruited over a planned 12-month enrollment period. The target sample size of approximately 10–15 participants was based on expected clinical throughput and feasibility considerations typical for pilot RCTs, rather than on a formal sample size calculation.

Eligible patients were identified by the attending clinical team following diagnostic confirmation and routine medical record screening according to site-specific standard care. Families were informed about the study shortly after diagnosis and invited to an in-person consultation with the study team. Subsequently, exercise therapists approached potential participants and their legal guardians in person to provide detailed information and disclose study aims and procedures.

Families who expressed interest received written study materials, and written informed consent from parents or legal guardians, in conjunction with age-appropriate assent from patients, were obtained prior to enrollment. Time between diagnosis, start of therapy, initial approach and final inclusion or exclusion was recorded.

#### 2.2.3 Planned randomization

We used a stratified randomization procedure with a minimization approach to ensure group balance in this small sample. After consultation with the Institute for Biostatistics and Mathematical Modeling at University Medicine Frankfurt am Main, independent research personnel generated a simple randomization scheme (1:1 ratio) and used sequentially numbered, opaque, sealed envelopes for allocation concealment (38). Corresponding envelopes were opened only after enrolled participants completed all baseline assessments. However, if new participants matched a previously enrolled patient based on gender and age group (10–13 years, 14–17 years), they were not randomized but paired to yield a minimum of six pairs from no more than 15 participants in total.

While exercise therapists administering training were aware of group allocation, patients and their families were not explicitly informed about their allocated arm but may have inferred it from the type of exercise completed. All other clinical personnel (e.g., physicians, nurses, nutritionists) and assessors (e.g., for motoric tests, sonography, blood sampling) were kept blinded.

### 2.3 Intervention and comparator

The present trial was designed to expand on usual care by implementing exercise training as early as induction therapy starts to specifically mitigate adverse effects of high-dose corticosteroids treatment. Accordingly, the intervention covered the first eight weeks of therapy for each patient, beginning on the day of initial corticosteroid administration, and replaced the usual-care exercise therapy offered at study site. The latter non-systematically introduces all pediatric oncology patients from the age of three and irrespective of the type of cancer to exercise therapy within the first few weeks of treatment. In line with national exercise therapy guidelines (39), usual-care training sessions are offered several times a week and highly individualized in intensity and contents depending on patients', availability, medical treatment, physical condition, exercise preferences and resources.

Participants randomized to the intervention group received a multimodal exercise program targeting aerobic and muscular strength components of physical fitness at moderate to vigorous intensity, whereas those allocated to the control group completed a sham exercise program with low-intensity activities. Thresholds for target exercise intensity were not disclosed to participants.

Irrespective of group allocation, exercise sessions were delivered 1:1 by one of three professional in-house exercise therapists, either face-to-face (e.g., in the clinic's gym) or remote (e.g., via video-call). Where appropriate, parents were present during exercise sessions but not necessarily involved. Following site-specific usual care and national exercise therapy guidelines (39) exercise sessions were scheduled individually with respect to patients' regular in-house appointments, clinical procedures and personal preferences. Typically, training took place between 9:00 a.m. and 5:00 p.m., alongside in- and outpatient visits, and both before and after clinical examination depending on patients' condition.

Both arms received one initial nutritional consultation to ensure that all participants were equally informed about potential side effects of corticosteroid treatment on body composition and metabolic health. Nutritional counselling lasted approximately 60 min including a semi-structured interview for nutritional assessment as well as age-specific dietary advice considering anthropometric changes related to high-dose corticosteroid treatment, gastrointestinal treatment-related symptoms, adequate protein intake and intake of sugar-sweetened beverages. Sufficient time was allocated to individual questions regarding food hygiene, storage, cooking, preparation, and food service in the clinical setting. Details on the procedure and contents of the nutrition counselling are provided as Supplementary Material.

#### 2.3.1 Intervention group: multimodal exercise

At the beginning of each session, patients were asked to depict their emotional and physical state on a poster. Therapists reasonably assigned or, if applicable, participants self-selected a warm-up routine, one of five pre-defined exercises per category, as well as a cool-down or free play in the end.

Exercises, routines or sport games, including potential variations to tailor them to participants' individual preferences, skill level, and condition were categorized by targeted muscles (i.e., core, upper, or lower extremity) and direction of motion (i.e., push or pull). The main part involved circuit training with two sets of five exercises, each with 40 s of time-under-tension, 20 s rest and a 2 min active rest between sets.

Perceived exertion was recorded after each session using the original Borg Rating of Perceived Exertion (RPE) scale [6 = very light; 20 = very, very hard; (40, 41)]. The target training intensity for metabolically relevant exercise was moderate to vigorous [RPE ≥ 12; (42, 43)].

#### 2.3.2 Control group: sham exercise

Control group patients also reported their current emotional and physical state before exercising. Then, therapists suggested, or participants self-selected up to five different low-intensity or relaxation exercises or games, with the first and last representing warm-up and cool-down, respectively. In the end, patients also report their perceived exertion using RPE. In contrast to the intervention group, intensity was supposed to be fairly light [Borg scale RPE ≤ 11; (42, 43)] to be not metabolically relevant.

#### 2.3.3 Protocol fidelity

To monitor study protocol fidelity despite inevitable diversity, therapists completed a session log after meeting a patient with both quantitative and qualitative characteristics (e.g., setting, content, duration, subjective intensity, reasons for refusal of participation or early termination). Other, off-protocol modifications were guided by valid and reasonable strategies to benefit intervention feasibility (e.g., increase exercise adherence, reduce harm or risks) and documented including their underlying rationale.

Sessions were postponed or cancelled if clinical evaluation revealed any medical condition that temporarily contraindicated physical activity or patients refused to participate despite verbal encouragement. Other contraindications included:

- Fever ≥38.0 °C or fever accompanied by systemic symptoms (e.g., chills, vertigo, pronounced fatigue).
- Acute infection or clinical signs of systemic illness (e.g., pneumonia, sepsis, unexplained tachycardia).
- Uncontrolled pain, new or worsening musculoskeletal symptoms, or inability to move safely.
- Severe gastrointestinal symptoms, including vomiting (not nausea) or clinically relevant diarrhea.
- Recent invasive procedures associated with bleeding risk or post-procedural symptoms (e.g., severe headache after lumbar puncture).
- Transfer to intensive care or any acute clinical deterioration requiring stabilization.

Whenever a temporary contraindication was present, patients remained enrolled in the study, and sessions were resumed once medical clearance was re-established. Activities were modified—but not necessarily cancelled—at thrombocyte counts between 10.000 and 30.000/mm^3^ or hemoglobin levels between 6.0 and 8.0 g/dL, depending on individual bleeding risk, clinical symptoms, and physician approval (44, 45).

Participants were classified as being treated as planned if they completed at least 80% of training at group target intensity. Arguably, patients do rarely exercise at a metabolically relevant level outside of supervised sessions, hence, hypothetical home-based training or physiotherapy was not controlled for as confounder.

### 2.4 Outcomes

#### 2.4.1 Recruitment and retention

After cessation of the trial, measures of availability, acceptance and attrition were used to evaluate feasibility of patient recruitment and retention. Cut-offs to determine whether continuation to a definitive trial is reasonable were predefined in accordance with previous studies (31, 46) including successful inclusion of 10–15 participants within 12 months and an overall consent rate of at least 80%. In addition, the number of dropouts was recorded alongside reasons given by patients or families for not consenting or withdrawing study participation.

#### 2.4.2 Implementation fidelity

The delivery and adherence to the intervention as planned was individually monitored throughout the intervention period, including the number and duration of exercise sessions completed overall and at target intensity of the allocated group. Participants were required to complete at least 80% of sessions offered with a minimum training volume of 48 min per week (i.e., 384 min in total equals 80%). Reasons for refusing participation or early termination were recorded. Furthermore, mean session duration and intensity per participant, as well as the proportion of sessions at target intensity were used to estimate exercise dosage, quality of delivery and group differentiation. Adherence to measurement was quantified by the timing and completion of outcome assessments. The number of and corresponding reasons for incomplete and delayed measures were noted.

#### 2.4.3 Harms and safety

While exercise therapists regularly documented adverse events of exercise interventions or assessments (and their presumed cause) according to the Common Terminology Criteria for Adverse Events [CTCAE (47, 48)], medical staff non-systematically checked for other potentially study related harms as part of standard care alongside therapy. In addition, serum markers commonly used as safety parameters for exercise were obtained in weeks of motoric testing (C-reactive protein, thrombocytes, hemoglobin, neutrophil granulocytes, creatine kinase, albumin).

#### 2.4.4 Subjective evaluation

Personnel involved (i.e., medical staff, assessors, exercise therapists) and participants received distinct questionnaires developed by the study team to subjectively evaluate the feasibility of the study and intervention. After a patient completed final exercise testing, the family was given a paper-pencil version to be returned on their next clinical appointment. Staff involved were asked to answer anonymously via an online survey tool (SoSci Survey, Version 3.7.06) after the end of the trial.

Patients' self-reported acceptability of the intervention was based on four questions with a total of 27 items containing closed questions or statements about the perceived demands, contents, and setting of exercise sessions to be rated on a five-point Likert scale (e.g., 1 = “I do not agree at all”, 5 = “I completely agree”). Accordingly, attitudes towards and perceived consequences of exercise were evaluated by patients' agreement to 14 and 22 items, respectively. Moreover, perceived effects of training were rated in two questions with 38 items using a six-point Likert scale. For any changes declared here, patients were asked to specify the duration of effects (e.g., short-term = “immediately after/for the same day of training” vs. long-term = “for longer than the day of training”).

Personnel involved were asked to comment on the information received about the study, communication with the study team, the tasks they adopted, and time spent on study duties. In addition, they evaluated additional expenses for themselves and participants (e.g., costs, time, other resources), as well as areas for improvement or concerns related to similar trials in the future. In addition, exercise session logs and individual perceptions of exercise therapists involved in training and testing were discussed and eventually synthesized to recurring themes. Questionnaires are available (in German only) upon request from the corresponding author.

### 2.5 Patient-level outcomes

#### 2.5.1 Physical performance and functional capacity

A multitude of outcomes was used to inform which physical domains and associated measures were most sensitive and feasible in the early phase of treatment. Primary measures involved lower muscle strength and endurance (five-times sit-to-stand test; 5-T STS), isometric hand grip strength, as well as cardiorespiratory fitness (original steep ramp test protocol on cycle ergometer; SRT). Complementing secondary assessments included psychomotor speed (finger tapping test), upper and lower body explosive power (medicine ball shot and standing long jump), and static balance (60 s single leg stance). An overview of outcomes with corresponding methodological and normative references if available and detailed descriptions of test procedures are available as Supplementary Material.

#### 2.5.2 Body composition

##### 2.5.2.1 Ultrasound measures of quadriceps muscle and visceral fat thickness

All measures were performed by a single operator trained in basic ultrasound measurements and blinded to group allocation using a single B-mode ultrasound system (Logiq S8 XDclear, GE Healthcare, Chicago, Illinois, USA). Two different array probes were employed depending on the anatomical region assessed: a high-frequency linear-array probe (ML6-15) was used for measurements of the quadriceps femoris muscle, while a low-frequency convex transducer (C2-9) was utilized for the assessment of intraabdominal adipose tissue to ensure sufficient tissue penetration.

Participants were positioned in a relaxed supine posture with legs extended and muscles relaxed throughout all assessments. Measurement sites were selected based on previous literature and pretesting experience (49–51). For intraabdominal adipose tissue measurements, the transducer was placed transversely approximately 1 cm above the umbilicus, targeting the region between the posterior side of linea alba and the anterior wall of the aorta. For quadriceps muscle thickness, the probe was positioned in an axial orientation perpendicular to the muscle belly, at a location halfway between the anterior superior iliac spine and the superior aspect of the patella, corresponding to the site of maximal muscle diameter.

Fixed machine settings were maintained across all scans (quadriceps muscle dB = 69, MHz = 15, Hz = 12–27; abdomen dB = 69, MHz = 6, Hz = 27–38), with only the scanning depth adjusted to accommodate individual anatomical variation. A liberal amount of water-soluble transmission gel was applied to minimize tissue compression and preserve the natural curvature of the skin. Duplicate measurements were acquired at each anatomical site. Two static images were captured under standardized conditions and muscle thickness measured directly using the ultrasound system's native software. If the variance between the two measurements exceeded 10%, additional images were obtained until two consistent values were achieved.

##### 2.5.2.2 Anthropometry

Patients' waist circumference, body height and weight were measured prior to motoric testing at three timepoints if no recent values were acquired in routine care and available from their medical record. Waist circumference was measured at the narrowest part on similar level to the midpoint between the highest point of the iliac crest and the last costal arch timed to exhalation. Waist-to-height ratio (WtHR) and body mass index (BMI) were calculated according to international standards (52).

$$\mathrm{WtHR}=\frac{\mathrm{Waist}\;\mathrm{circumference}\;[\mathrm{cm}]}{\mathrm{Body}\;\mathrm{height}\;[\mathrm{cm}]}$$

$$\mathrm{BMI}=\frac{\mathrm{Body}\;\mathrm{weight}\;[\mathrm{kg}]}{{(\mathrm{Body}\;\mathrm{height})}^{2}[cm^{2}]}$$

#### 2.5.3 Metabolic health

Key metabolic health parameters regularly monitored during acute therapy in childhood cancer include short- and long-term blood glucose, as well as serum insulin levels (5, 53). Triglycerides and cholesterol were obtained as additional markers to identify early metabolic dysregulation (54, 55). Blood sampling was administered by medical staff blinded to intervention allocation and integrated into standard care.

##### 2.5.3.1 Blood glucose and insulin

Fasting insulin, as well as fasting and postprandial (i.e., approximately two hours after meal) blood glucose levels were obtained at baseline, in week one, week four and week eight of therapy to derive Homeostatic Model Assessment for Insulin Resistance [HOMA-IR; original calculation as in Ref. (56)] as indicator of insulin resistance if values are above the 90th percentile of age-specific reference values (54).

$$\frac{\mathrm{HOMA}-\mathrm{IR}=\mathrm{Fasting}\;\mathrm{insulin}\;\left[\frac{\mu U}{\mathrm{mL}}\right]\;*\;\mathrm{fasting}\;\mathrm{glucose}\;\left[\frac{\mathrm{mg}}{\mathrm{dL}}\right]}{405}$$

Moreover, the overall incidence of hyperglycemia [i.e., more than two blood glucose tests above 200 mg/dL; (5)] and unusual bowel movements were monitored throughout the trial.

##### 2.5.3.2 Glycated hemoglobin

Hemoglobin A1c (HbA1c) is a parameter commonly used in the diagnosis and control of diabetes mellitus to measure average blood glucose levels over the past 90 days (57), typically expressed as a percentage. Higher values represent more glucose being attached to hemoglobin protein in erythrocytes (57). After twelve weeks of therapy, patients' HbA1c is used to check for irregularities and discrepancy between different measures of blood glucose levels.

### 2.6 Statistical analysis

Quantitative evaluation of study feasibility was based on rates (e.g., drop-outs, adverse events) and ratios (e.g., percentage of completed exercise sessions, missing/incomplete tests). Subsequently, dichotomous variables are derived from comparing obtained results to prespecified criteria (e.g., achieved vs. not achieved) and aggregated to a decision about the potential for a definitive trial in the future (see Supplementary Material). In addition, subjective feedback from patients and personnel involved was used to identify common themes related to the perception and acceptability of trials in the early treatment phase (thematic analysis).

Demographic information from all participants was obtained to characterize the sample and contrast intervention and control group at baseline. Due to small sample size, baseline differences between groups were not calculated but explored descriptively (data tables in Supplementary Material). Primary outcomes at t0 were descriptively compared to gender- and age-specific healthy references, as well as diagnostic criteria of health-related issues (e.g., obesity, metabolic syndrome, insulin resistance). Corresponding outcome trajectories are depicted as absolute and relative changes over time using all available data, without imputation of missing values. If applicable, Z-score differences from reference values were calculated. Inferential analyses including (non-) parametric intra- and intergroup comparisons for outcomes on patient-level were not conducted due to lower-than-expected eligibility and recruitment rates.

Given the small sample size, measures of central tendency and dispersion only include median and interquartile range (IQR). However, overall mean and standard deviation for each outcome is available in Supplementary Material. Calculations were performed using Microsoft Excel (Microsoft Corp., Redmond, Washington, USA; Version 2511), and IBM SPSS Statistics [IBM Corp., Armonk, New York, USA; Version 31.0.0.0 (117)]. Figures were generated with Microsoft Excel and PowerPoint (Microsoft Corp., Redmond, Washington, USA; Version 2511).

## 3 Results

From July 2024 to July 2025, nine adolescents newly diagnosed with cancer were eligible. Three patients declined participation for various reasons related to inherent components of the trial. In total, this resulted in a sample of six patients, with the last completing their final 12-week follow-up in August 2025. Of those who consented, four participants were individually randomized and two were matched to a previously enrolled patient. The trial ended after planned recruitment over 12 months was finalized. Figure 1 provides an overview of participant screening, enrollment, allocation, follow-up, and inclusion in the descriptive analysis according to CONSORT reporting guidelines (58).

**Figure 1:**
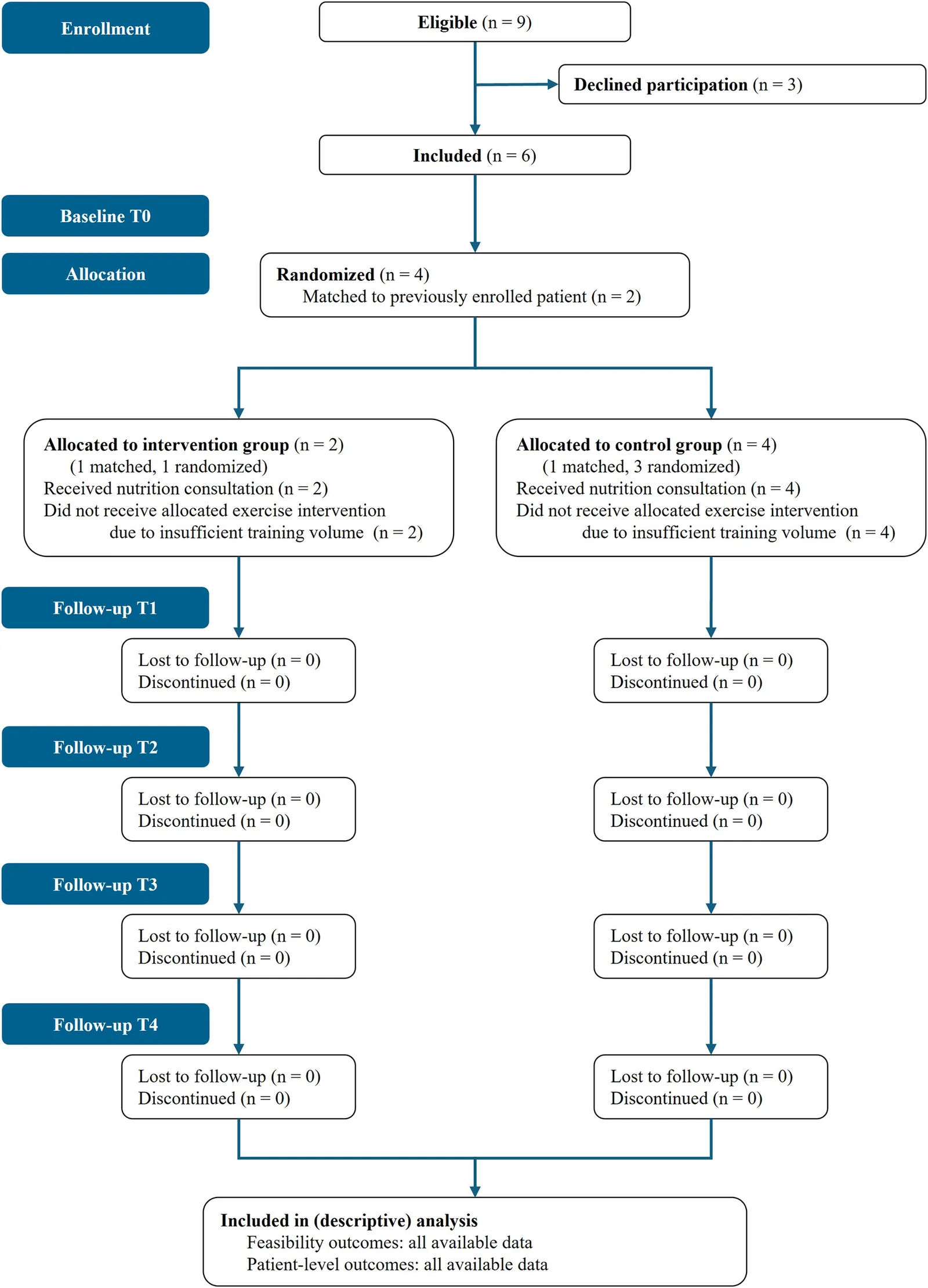
CONSORT study flowchart. Overview of participant screening, enrollment, allocation, follow-up and inclusion in descriptive analysis according to CONSORT guidelines (58).

Anthropometric and clinical baseline characteristics for the overall sample and by study group are presented in Table 1. Distribution of age, gender, BMI, WtHR, diagnosis (leukemia vs. lymphoma), and treatment protocol was balanced between groups. Patients received dexamethasone, prednisone and/or prednisolone based on different treatment protocols in current clinical trials and guidelines (37). Details on the dosage and administration of corticosteroids alongside the intervention are available as Supplementary Material. Moreover, patients stated no pre-existing comorbidities at baseline.

**Table 1:** Participants' anthropometric and clinical characteristics.

Characteristic	Overall, *n* = 6	Intervention, *n* = 2	Control, *n* = 4
	Median (Q1–Q3)	Min–Max	Median (Q1–Q3)
Age (years)	14.0 (13.3–14.5)	14.0–14.0	14.0 (11.8–15.5)
Females	3 (50%)	1 (50%)	2 (50%)
Height (cm)	157.3 (155.0–177.8)	157.5–171.1	156.5 (153.0–170.9)
Weight (kg)	49.5 (44.8–56.2)	44.0–56.4	49.5 (46.0–51.5)
BMI (kg/m^2^)	19.7 (17.5–20.3)	17.7–19.0	19.7 (17.5–20.4)
WtHR	0.44 (0.41–0.49)	0.41–0.42	0.46 (0.40–0.52)
Right hand dominant	4 (67%)	1 (50%)	3 (75%)
Diagnosis			
ALL	3 (50%)	1 (50%)	2 (50%)
HL	1 (16.7%)	1 (50%)	—
NHL	2 (33.3%)	—	2 (50%)
Prescribed corticosteroid			
Dexamethasone	4 (66.6%)	—	3 (75%)
Prednisone	1 (16.7%)	1 (50%)	—
Prednisolone	4 (66.6%)	1 (50%)	3 (75%)
Start of treatment after diagnosis (days)	6.5 (1.0–13.5)	1.0–8.0	6.5 (1.3–12.5)
Enrolment after start of treatment (days)	5.0 (2.3–6.3)	5.0–6.0	4.0 (0.8–5.8)
Comorbidities	—	—	—

Reasonably rounded median and interquartile or group ranges, as well as the absolute and relative frequency are presented for each characteristic within the sample and each group. ALL, acute lymphocytic leukemia; BMI, body mass index; HL, Hodgkin lymphoma; NHL, non-Hodgkin lymphoma; Min, minimum; Max, maximum; Q1, first quartile; Q3, third quartile; WtHR, waist-to-height ratio.

### 3.1 Recruitment and retention

Feasibility outcomes and predefined cut-offs are presented in Table 2. Targets of study consent were not achieved as only nine adolescent patients were eligible, of which one third did not consent to participate in the study. Reasons given were low interest in and motivation for exercise, difficulties adjusting to diagnosis and treatment, and general skepticism about study participation and additional, though non-invasive, diagnostic procedures. Still, these patients adopted exercise therapy as per standard care in later stages of therapy.

**Table 2:** Overview of feasibility outcome measures and results.

Domain	Measure	Metric calculation	Trial results	Cut-off
Recruitment	Availability of eligible patients	No. of patients fulfilling inclusion criteria	9	10–15
	Acceptance across eligible patients	Proportion of patients enrolled after approach	67%	80%
		Reasons for declined study participation	Low interest in sport Difficulties adjusting Skepticism about study	—
Retention	Attrition rate	Proportion of patients dropped out after enrollment (reasons for drop-out)	0 (-)	—
Implementation fidelity	Adherence to intervention	Proportion of exercise sessions completed vs. sessions offered	51%	80%
		Exercise volume (minutes)	185 (102.5–325.0)	≥384
		No. of weeks without supervised exercise per participant	2.5 (0.8–3.5)	—
		Reasons for refused participation or termination of exercise	Experiencing symptoms Organizational issues Time constraints Contraindications	—
	Training dosage	Mean exercise session duration (minutes)	30.5 (28.1–32.8)	≥20
		Mean exercise session intensity (RPE)	IG 13.4 (10.9–13.4)	IG > 11
			CG 8.4 (8.0–12.2)	CG ≤ 11
		Proportion of exercise sessions completed at target intensity	89.7%	80%
	Adherence to measurement	Proportion of incomplete measurements	30.3%	—
		Proportion of delayed measurements	71.2%	—
		Reasons for incomplete or delayed measurements	Low interest in sport Difficulties adjusting Skepticism about study	—
Harms and safety	Adverse events	Incidence of adverse events (reasons, CTCAE classification)^1^	0 (-)	—
Subjective evaluation	Questionnaire	Self-report, patients (and/or) parents	Positive experience Perceived as beneficial	—
	Questionnaire	Self-report, involved personnel	Acceptable perceived fit to organizational structures Challenging but valuable to implement exercise testing and intervention	—

Reasonably rounded absolute counts and proportions of primary and secondary feasibility outcomes are presented in non-shaded and shaded cells, respectively. If applicable, median and interquartile ranges are given, as well as key points from qualitive data assessment. ^1^Common terminology criteria for adverse events (47). CG, control group; IG, intervention group; No., number; RPE, rating of perceived exertion.

Although all patients were screened within the first three days after diagnosis, only one participant was recruited at this predefined time. Instead, study inclusion was achieved after a median of 6.5 days after diagnosis (IQR: 1.0–13.5), corresponding to 5.0 days after start of treatment (IQR: 2.3–6.3). Yet, attrition was acceptable since no patient dropped out or was lost to follow-up throughout the trial.

### 3.2 Implementation fidelity

Of all exercise sessions offered, patients agreed to participate in 51%, with individual rates ranging from 18 to 73%. In conjunction with the total exercise volume per participant (median 185 min, IQR: 102.5–325.0), training adherence failed to attain pre-specified thresholds. Although online training was offered as equivalent alternative, particularly if exercise sessions could not be conducted on-site, only one was held remotely. Throughout the study period, one patient received additional physiotherapy in terms of breathing exercises.

With a median training frequency of 0.7 sessions per week (IQR: 0.4–1.4), patients did not exercise for about two and a half weeks, with lowest rates of adherence in weeks three and seven. The most common reasons why participants refused to exercise were experience of symptoms (mentioned 26 times), most commonly fatigue (65.4%) and pain (27.9%), as well as organizational issues and time constraints (mentioned 12 times) such as other appointments in the clinic (50%) or family plans (29%). Also, pre-defined contraindications, though mostly temporary and in conjunction with other symptoms, were prevalent throughout the intervention period in all patients and detained them from training (mentioned 15 times). Omitted sessions were declined for more than a single reason in 55%. Still, patients of both groups attained their respective target exercise intensity in more than 80% of sessions completed. Duration of individual training sessions was similar in both groups (median of 27.0 and 31.3 min in intervention and control group, respectively), whereas self-reported intensity differed as prescribed per protocol.

Overall, outcome measures were frequently delayed (71.2%, i.e., not administered in predefined week) and/or incomplete (30.3%, i.e., conducted but with items missing). Blood sampling failed most frequently, compromising data completion of metabolic health, whereas exercise testing and sonography were more affected by delays. In terms of blood sampling and sonography, this was mostly due to organizational difficulties fitting study procedures into patients' clinical schedules and coordinating medical personnel. Occasionally, patients also did not comply with regulation of food intake for testing and claimed fasting to be somewhat strenuous. Accordingly, parameters that could not be obtained most frequently were fasting insulin and HOMA-IR (8 times, 33.3%).

Physical performance and functional capacity assessments were demanding for patients (median duration of 39.2 min, median Borg RPE 14.0), up to the point that they were unable or unwilling to complete additional training afterwards (mentioned 14 times). Moreover, testing was repeatedly postponed or modified due to patients' reluctance, lack of exercise capability/tolerance, or safety concerns. Across all times of measurement, standing long jump had the highest proportion of missing data (4 times; 22%). Moreover, 58% of physical follow-up assessments at t2 and t3 had to be conducted by the study team (i.e., not blinded to group allocation) as blinded assessors were not available.

### 3.3 Harms and safety

Patients did not experience any adverse events related to the intervention. However, four exercise sessions were terminated early because of patients' fatigue or lack of motivation to continue.

### 3.4 Subjective evaluation

All patients rated their participation in the exercise program during their first eight weeks of treatment as positive experience (median 4.5, IQR: 4.0–5.0; 5 = “very positive experience”). Though training was reported to be somewhat physically and mentally demanding, all patients were satisfied with its frequency and duration (i.e., “neither too many/long, nor too few/short”). Session contents were considered fun (median 5.0, IQR: 3.8–5.0) and tailored to individual abilities (median 4.0, IQR: 4.0–5.0). In terms of exercise setting, however, some participants preferred to exercise on their own but with therapist's plan or, if supervised, as online training.

Overall, perceived fit with organizational structures evaluated by medical and nursing staff was good but required continuous interprofessional communication. On that note, information about study aims and procedures prior to commencement, as well as availability of study team to answer questions were deemed essential. The personnel involved stated low additional efforts and did not express any concerns or objections regarding future studies apart from their generally limited resources.

As part of the study team, exercise therapists conducting exercise sessions perceived the trial and intervention to be both challenging and meaningful. Challenges were reported to mainly arise from the first days following diagnosis being emotionally and logistically turbulent for families. The excessive amount of information and little time to process and adapt to the new situation may cut back on families feeling competent enough to make an informed decision about study participation. Moreover, difficulties conducting assessments and training were reported as they were competing with other diagnostic appointments in the clinic throughout the first two weeks. Moreover, having an intravenous cannula limited some physical assessments. On the contrary, early assessments were perceived to support learning about the patient's objective physical capacity, subjectively perceived physical limits, and the kind and extent of motivational, emotional and physical support and autonomy a patient needed and preferred. Initiating exercise interventions as early as the first week after diagnosis was considered to facilitate the development of trust in the patient–therapist relationship, as the latter was better able to relate to the patient's physical trajectory and gain a clearer understanding of the patient's pre-diagnosis exercise habits and physical identity.

### 3.5 Patient-level outcomes

#### 3.5.1 Physical performance and function capacity

Physical assessments at baseline were administered after a median of seven days of treatment. Table 3 presents baseline values of the overall sample for primary and secondary outcome metrics including absolute and relative differences from healthy reference populations. One week into induction therapy, patients' mean scores were inferior across all primary metrics. With respect to secondary outcomes, the majority of participants also scored below the 50th percentile of age- and gender-specific norms available. However, the wide range of values, confidence intervals and high standard deviations marked a small, yet heterogeneous sample.

**Table 3:** Physical performance and functional capacity outcomes at baseline.

		Sample	Healthy reference		
Measure	*N*	Median (IQR)	Δ*M* ± SD	Δ*Z* ± SD	Inferior to norm^1^
5-T STS (s)	6	8.5 (7.7–11.6)	2.7 ± 2.4^a^	2.3 ± 2.0^a^	5 (83%)*
Grip strength D (kg)	6	20.8 (13.1–27.8)	−9.4 ± 8.6	−1.5 ± 1.4	6 (100%)*
Grip strength ND (kg)	6	18.2 (13.1–24.8)	−10.8 ± 8.4	−1.8 ± 1.6	6 (100%)*
SRT peak power (w)	6	130.5 (111.5–178.8)	−150.8 ± 64.3	−3.8 ± 1.2	6 (100%)*
SRT peak HR (bpm)	6	141.5 (120.5–161.0)	−39.7 ± 23.2	−4.0 ± 2.3	6 (100%)**
MBS (m)	5	4.2 (2.1–5.1)	n/a	n/a	n/a
SLJ (cm)	5	106.0 (94.0–144.3)	n/a	n/a	5 (100%)*
SLS (errors)	5	9.0 (5.5–25.0)	n/a	n/a	2 (40%)*
TAP (count)	6	56.7 (51.5–62.3)	−3.0 ± 5.4	−0.6 ± 1.0	3 (50%)*

Reasonably rounded sample median and interquartile ranges are presented for primary and secondary outcomes in non-shaded and shaded cells, respectively. Mean differences from healthy reference populations are shown including standard deviations and z-scores. *Age- and sex-specific norms. **Sex-specific norms from an 8–19-year-old cohort. ^1^Mean, if not available, median. 5-T STS, five-times sit-to-stand test. ^a^Positive values indicate that healthy peers performed the test faster (i.e., better). D, dominant hand; HR, heart rate; IQR, interquartile range; n/a, not applicable; ND, non-dominant hand; MBS, medicine ball shot; SLJ, standing long jump; SLS, single leg stance; SRT, steep ramp test protocol; TAP, finger tapping.

Deviations were largest for peak power and peak heart rate during SRT (Δ*Z* ± SD = −3.81 ± 1.25; Δ*Z* ± SD = −3.97 ± 2.32), as well as duration of the 5-times sit-to-stand (Δ*Z* ± SD = 2.25 ± 2.03; here, positive values indicate that the sample was slower than reference). A relatively small difference from healthy norms was observed in finger tapping.

Figure 2 presents primary outcomes as boxplots for each measurement timepoints for the overall sample. Within 4 weeks after commencement of therapy, all primary measures of physical performance and functional capacity declined independent of the allocated group. The most pronounced relative decline occurred in the 5-T STS with a about 60% slower execution at t2 compared to baseline. Similarly, SRT performance deteriorates by means of a higher peak heart rate in conjunction with lower peak power. However, the latter tends to recover to nearly baseline level between follow-ups at t2 and t3, while heart rate remains elevated. Most consistently among patients, hand grip strength reduced by roughly 25% within the first four weeks and continued to decline over the overall intervention period. Secondary outcomes show no such distinct pattern, but markedly low retention rates in standing long jump.

**Figure 2:**
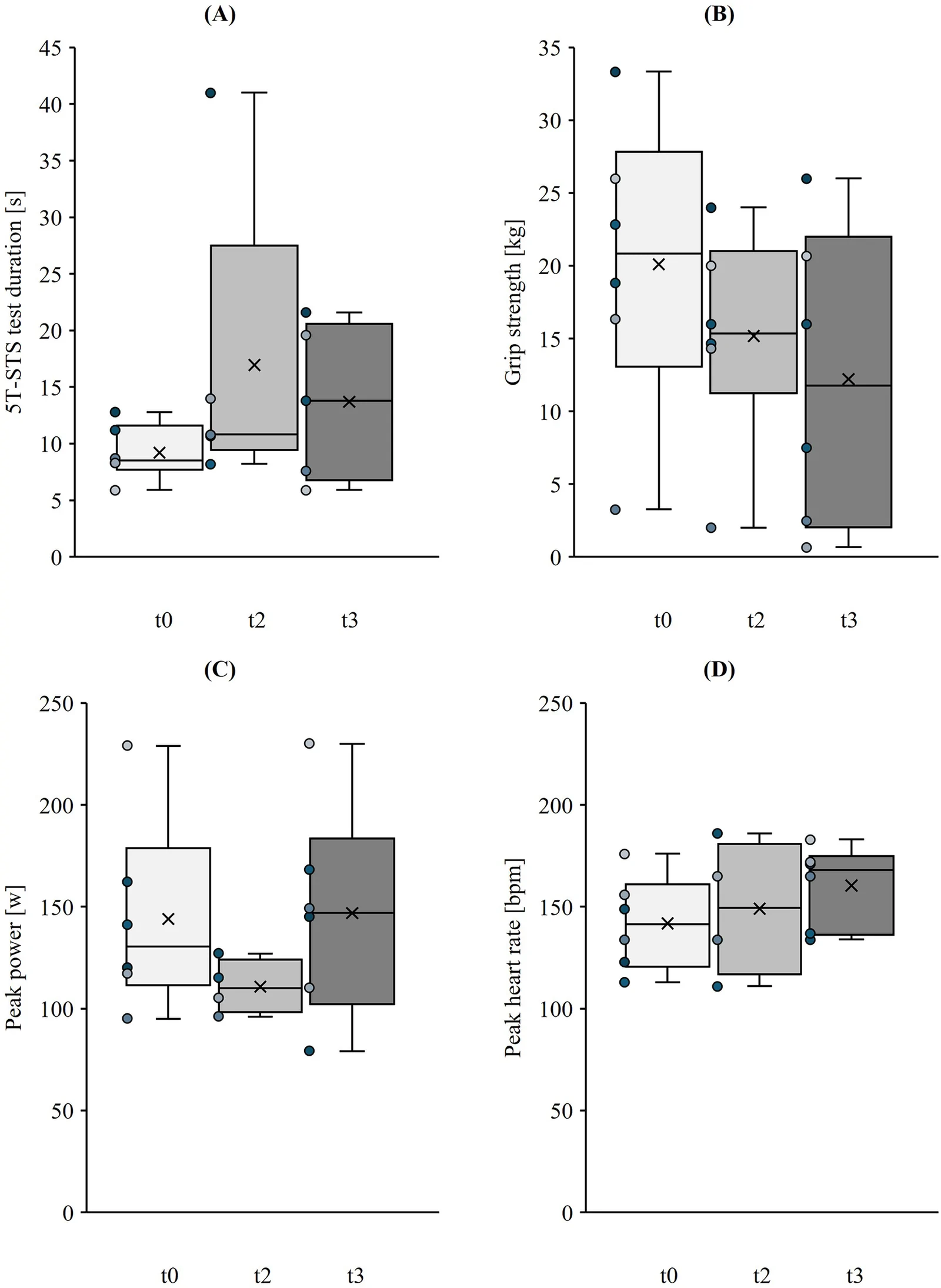
Primary outcomes of physical performance and functional capacity over time. Boxplots of primary outcomes including sample median (⸺), mean (x), range (minimum and maximum whiskers), and individual data points (●). **(A)** 5-times sit-to-stand test, higher values indicate slower execution. **(B)** Isometric hand grip strength in kilogram, dominant hand. **(C)** Steep ramp cycle ergometry peak power in watts. **(D)** Steep ramp cycle ergometry peak heart rate in beats per minute.

#### 3.5.2 Body composition

Initial anthropometry and sonography were conducted up to fifteen days following the start of therapy (ranging from day one to eight and day five to fifteen, respectively). Baseline body composition of the sample is depicted in Table 4. Compared to healthy pediatric reference data, one of six patients was beyond normal range WtHR (i.e., >0.49 when screening for abdominal obesity) but not BMI (i.e., >P90 or P97 for overweight or obesity, respectively). Mean quadriceps muscle at baseline was comparable with age-specific norms, yet individual differences were highly variable. Visceral fat thickness was slightly higher than sample data from the original method reference (*n* = 9, aged 10.3 ± 2.3 years, <85th BMI percentile). Over time, sonographic measures demonstrate highly variable changes in visceral fat and quadriceps muscle thickness, starting with a decline in a similar manner over the first four weeks of therapy (Figure 3). In contrast, between t2 and t3, abdominal fat thickness increases to approximately initial magnitude, whereas quadriceps muscle thickness further diminishes. However, these changes do not transfer to secondary measures, demonstrating rather constant WtHR and BMI scores across the period of intervention in all patients.

**Figure 3:**
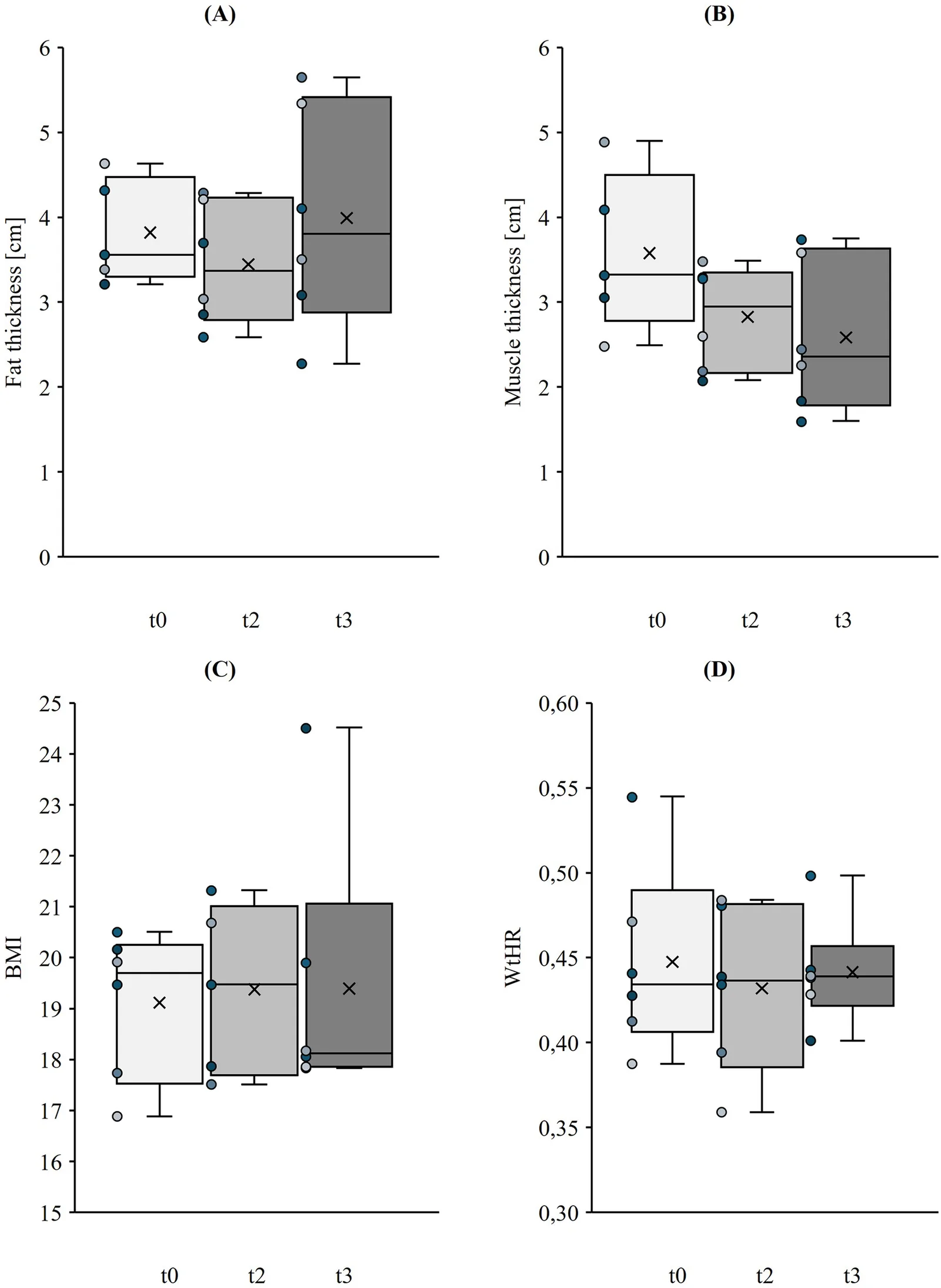
Body composition and anthropometric outcomes over time. Boxplots of primary outcomes including sample median (⸺), mean (x), range (minimum and maximum whiskers), and individual data points (●). Ultrasound-derived **(A)** visceral fat thickness and **(B)** quadriceps muscle thickness, in cm. **(C)** Waist-to-height ratio. **(D)** Body mass index.

**Table 4:** Body composition and anthropometric outcomes at baseline.

		Sample	Healthy reference		
Measure	*N*	Median (IQR)	Δ*M* ± SD	Δ*Z* ± SD	Diagnostic cut-off
VFT (cm)	5	3.6 (3.3–4.5)	1.1 ± 0.6*	0.4 ± 1.6	1 (17%) > P95^a^
QMT (cm)	5	3.3 (2.8–4.5)	0.1 ± 1.1**	0.03 ± 2.4	n/a
BMI	5	19.7 (17.5–20.3)	n/a	n/a	0 (0%) > P80^b^
					0 (0%) > P95^b^
WtHR	6	0.44 (0.41–0.49)	n/a	n/a	1 (17%) > 0.49^c^

Reasonably rounded sample median and interquartile ranges are presented for primary and secondary outcomes in non-shaded and shaded cells, respectively. Mean differences from healthy reference populations are shown including standard deviations and z-scores. *Non-specific sample data from method reference. **Gender-mixed norms, specific to age-groups (11–12 years, 13–16 years). ^a^Metabolic syndrome (76). ^b^overweight and obesity (66). BMI, body mass index. ^c^(Abdominal) obesity (52). n/a, not applicable; QMT, quadriceps muscle thickness; VFT, visceral fat thickness; WtHR, waist-to-height ratio.

#### 3.5.3 Metabolic health

Baseline blood samples depicted in Table 5 and Figure 4 were obtained within the first eight days of therapy (after a median of 2.5 days) but required more than a single day of assessment to be completed in all cases. Despite considerably high variability, patients' fasting and postprandial glucose levels were within healthy reference range at baseline. Fasting insulin and corresponding HOMA-IR were measured in only two to four out of six participants at baseline and one week thereafter, hence, their presented levels—though elevated to the extent of the 95th percentile of age-specific references—were not representative of the overall sample. Secondary outcomes including triglycerides, total and LDL-cholesterol demonstrated elevated values whereas reduced HDL-cholesterol was apparent across the sample, some to the extent of diagnostic thresholds for hypertriglyceridemia and dyslipidemia.

**Figure 4:**
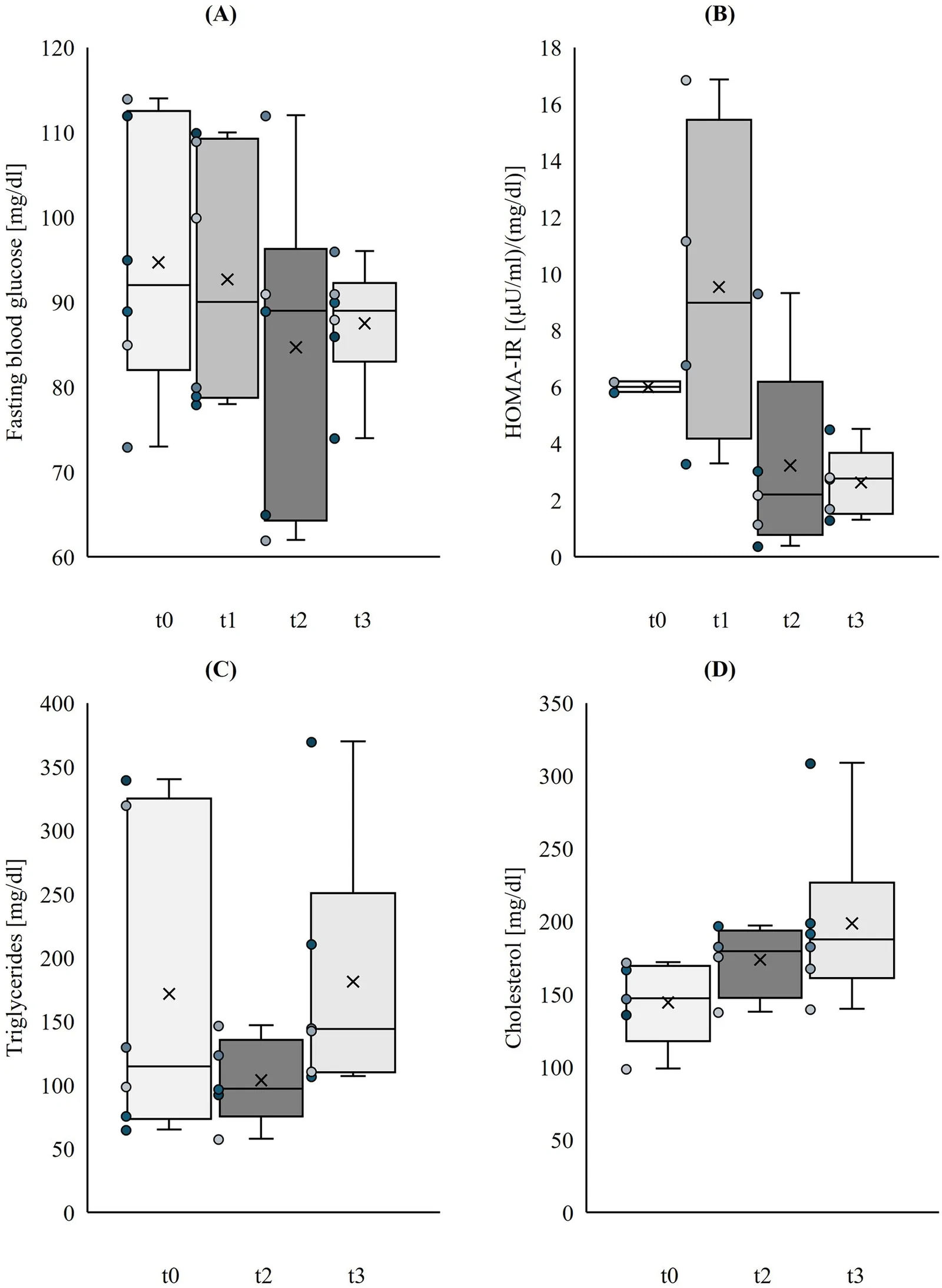
Metabolic health outcomes over time. Boxplots of primary outcomes including sample median (⸺), mean (x), range (minimum and maximum whiskers), and individual data points (●). Ultrasound-derived **(A)** Fasting blood glucose levels in mg/dL. **(B)** HOMA-IR **(C)** Triglycerides in mg/dL. **(D)** Total cholesterol in mg/dL.

**Table 5:** Metabolic health outcomes at baseline.

		Sample	Healthy reference		
Measure	*N*	Median (IQR)	Δ*M* ± SD	Δ*Z* ± SD	Diagnostic cut-off
PP BG (mg/dL)	5	126.0 (110.0–136.0)	n/a	n/a	0 (0%) ≥ 200^a^
					1 (33%) < 140^b^
F BG (mg/dL)	6	92.0 (82.0–112.5)	6.3 ± 15.8*	0.8 ± 2.0	0 (0%) ≥ 126^a^
					1 (17%) > 110, ≤125^b^
F insulin (μU/mL)	2	24.3 (-)	13.4 ± 4.1*	2.3 ± 0.7	2 (100%) > P9°^c^
HOMA-IR [(μU/mL)/(mg/dL)]	2	6.0 (-)	3.9 ± 0.1*	3.1 ± 0.3	2 (100%) > P9°^c^
TG (mg/dL)	6	114.5 (73.3–325.0)	156.1 ± 125.0*	17.9 ± 14.0	2 (33%) ≥ 300, ≤500^d^
TCH (mg/dL)	5	147.0 (117.5–169.5)	−12.7 ± 26.4**	−0.4 ± 0.8	1 (20%) ≥ 170, <200^e^
HDL-C (mg/dL)	5	35.9 (24.1–40.1)	−21.0 ± 9.0**	−1.5 ± 0.7	4 (80%) < 40^e^
LDL-C (mg/dL)	5	86.0 (48.7–104.5)	−24.5 ± 28.9**	−1.1 ± 1.1	1 (20%) ≥ 110, <129^e^

Reasonably rounded sample median and interquartile ranges are presented for primary and secondary outcomes in non-shaded and shaded cells, respectively. Mean differences from healthy reference populations are shown including standard deviations and z-scores. *Gender-mixed norms, specific to age groups (11–15 years, 16–23 years). ** = Sex-specific norms from age groups (10–13 years, 14–17 years). ^a^Hyperglycemia (5); diabetes (77). ^b^Impaired fasting glucose. BG, blood glucose. ^c^Insulin resistance (54). ^d^Grade 2 hypertriglyceridemia (47). ^e^Dyslipidemia (78). F, Fasting; HDL-C, High-density cholesterol; HOMA-IR, homeostatic model assessment for insulin resistance; IQR, interquartile range; LDL-C, low-density cholesterol; n/a, not applicable; PP, postprandial; TCH, total cholesterol; TG, triglycerides.

Due to missing longitudinal data, insulin resistance was excluded from preliminary analysis of outcome trajectories. Yet, cross-sectionally, only one patient demonstrated fasting insulin and HOMA-IR above age-specific cut-off levels at t3. On an intra-individual level, four patients exhibited reductions in HOMA-IR greater than 2.0 between two timepoints throughout the intervention, which were formerly noted as measurable improvement (18). Available data regarding fasting and postprandial blood glucose indicate no profound changes over time beyond high variability between subjects. After twelve weeks of therapy, also HbA1c is within normal range (median 5.5, IQR: 5.4–5.7). Regarding secondary measures of metabolic health, already elevated levels of triglycerides and cholesterol at baseline accumulated further towards t3.

#### 3.5.4 Intervention effects

Low consent rate and training adherence in the exercise intervention group did not allow for exploration of intervention effects on physical performance, functional capacity, metabolic health, or body composition.

## 4 Discussion

This pilot study examined the feasibility of a combined exercise and nutrition intervention and explored preliminary changes in physical function, body composition, and metabolic health during the first eight weeks of treatment in children and adolescents with leukemia or lymphoma undergoing high-dose corticosteroid induction therapy.

Principal findings suggest that the delivery of supervised exercise and repeated testing in this context is safe (i.e., without intervention-related adverse events), but feasibility outcomes did not meet pre-specified thresholds for consent rate and training adherence (67% and 51% achieved, respectively vs. 80% targeted). Therefore, results did not allow for continuation to a definitive trial without substantial changes to the protocol.

Although the small sample size (*n* = 6) limits generalizability and precludes formal efficacy analyses, physical performance and functional capacity outcomes suggested intermittent deconditioning in newly diagnosed pediatric patients compared to age- and sex-specific reference data at baseline and throughout the intervention period. Despite considerable variability, anthropometric and sonographic measures indicate mostly normal body composition at baseline, while metabolic markers showed erratic unfavorable patterns in a subset of patients.

### 4.1 Feasibility of recruitment and retention

The trial revealed substantial challenges in recruiting newly diagnosed patients to a complex exercise–nutrition intervention during early induction therapy. Availability of eligible patients at the given site was slightly lower than expected (9 vs. 10–15 newly diagnosed within 12 months). Also, 67% consent rate in the first few days after diagnosis (IQR: 2.3–6) was lower than previously reported by means of refusal [21 ± 16%; (30)] or recruitment rates [28.6 to 85.7%; (24)] in similar settings. Yet, all patients who initially refused did take part in supervised exercise training of usual care later on.

This pattern, taken together with the given reasons for declined participation, underscores that the early diagnostic and treatment phase is emotionally and logistically demanding for patients and families and requires careful consideration of the approach and timing to obtain informed consent. Besides their initial coping strategies and confidence to comply with the new situation, patients' and parents' decision making is governed by individual attitudes towards medical settings, clinical trials (59) and supportive care (28). Thus, a “recurring face” and continuous daily visits with smaller portions of information may refine general strategies to enhance recruitment (60, 61) and make education about exercise in general and study participation specifically more digestible (62, 63).

Still, approaching families early may allow more time to build trust and address individual concerns in a critical phase, which in turn can increase approval of subsequent counselling or interventions. In that sense, retention among those who consented was excellent compared to earlier studies [ranging from 52.4 to 97%, with 23 ± 12% mid-trials exclusions reported on average; (24, 30)]. All participants completed follow-ups and none withdrew from the study, even though fluctuating health status and periods of reduced well-being frequently limited patients' ability to engage in physical training, suggesting that once families agree to participate, continued involvement is acceptable and compatible with routine care.

### 4.2 Feasibility of intervention delivery and adherence

Implementation of the exercise program as planned proved challenging, as overall adherence was considerably lower than in previous studies [64.3% on average, ranging from 45.8 to 80%; (24)] About half of the sessions omitted were declined for more than a single reason, including disease- or therapy-related symptoms such as fatigue and pain, organizational barriers related to clinical procedures or appointments, limited exercise capacity after physical testing and medical contraindications. Although similar objections to exercise have been identified at other times during and after acute treatment (24, 30), their high prevalence and severity are likely to reflect the specific challenges of early therapy phases involving high-dose corticosteroids. Hence, newly diagnosed patients may prioritize other interests, for instance, symptom management and emotional adjustment, over structured physical activity and nutritional counseling (28).

In the present trial, incorporating flexible scheduling and offering home-based or digital sessions as proposed earlier (28, 31, 64), could not effectively compensate for missed in-hospital visits. Consequently, future trials focusing on pediatric patients with leukemia or lymphoma may need to accept a low achievable dose at early stages of therapy, use training schedules with gradual uptake of exercise volume, including symptom-adapted or very low intensity options during difficult weeks, or prolong the intervention period to the extent possible.

Notably, when sessions did take place, patients in both groups were generally able to reach their target intensity in more than 80% of sessions, as well as the prespecified session duration of about 30 min. This is consistent with existing literature (30) and suggests that the planned content, intensity, and mode of the supervised sessions were feasible for most patients on days when they were clinically stable. Thus, limited training adherence rather relates to how frequently patients were fit and available to train but less to the exercise dosage *per se*. In addition to substantial between-subject variability, lower training adherence seen in the intervention arm than in controls (27% vs. 60%) may be due to expected training intensity.

In line with the majority of studies in the field (24, 30, 48), the intervention is deemed to be safe in the absence of adverse events related to exercise. Also, based on subjective evaluations of families and therapists, the exercise program seemed acceptable in terms of the risk, demands and benefits perceived. In accordance with other intervention studies (25), patients declared to experience a variety of positive effects after supervised exercise, despite temporary contraindications or other objections to participate in training. Designing the intervention details in a participatory process together with adolescents may have helped to ensure exercise was perceived positively early on during acute treatment. From therapist's perspective, early exercise intervention was considered to support building a patient–therapist relationship (65) while testing gave insights into potential deficits, feasible training options, and the patient's physical trajectory (34).

Conducting structured physical assessments shortly after diagnosis was of great help to individualize future exercise sessions. Balance and functional mobility assessments helped to detect risk of falling or need for assistive devices for safe ambulation and participation in the intervention, while assessing the capacity to perform jumps supported evaluation whether patients can safely engage in coordinatively complex sports that require weight bearing on a single limb, running or rapid change of direction. Engaging patients in assessments focused on cardiovascular function early after diagnosis was helpful to initiate education about declines in hemoglobin and to learn about subjective experiences of exhaustion and the adolescent's body awareness. However, findings also suggest that the integration of systematic longitudinal assessment in an already complex clinical setting is ambitious and requires adequate structural, organizational, and personal resources.

### 4.3 Exploration of patient-level outcomes and assessment

Structured physical assessments at the start of therapy have been proposed to detect early limitations and need for intervention in pediatric patients newly diagnosed with cancer (34, 35). In fact, baseline results resemble findings from a growing body of literature demonstrating impairments of physical performance and functional capacity shortly after diagnosis (1, 2). Although assessments were administered at a median of one week after treatment initiation, thus, earlier than in the majority of previous studies [i.e., 7 to 31 days of therapy; (2)], baseline results are arguably confounded by joined drivers of early deconditioning including a symptomatic course of hematologic malignancies prior to treatment, recent infections, comorbidities, physical inactivity, hospitalization, and/or early-onset medical treatment effects. Thus, assessing physical performance and functional capacity may be worthwhile to detect deterioration but cannot be used to reliably infer their etiology or clinical significance.

At baseline, participants' anthropometric indices were within normal ranges (52, 66), accompanied by a largely normal quadriceps muscle thickness in sonography (50, 67). On one hand, this contrasts previous notions that children with cancer start treatment with significantly lower muscle mass than healthy peers (1). On the other hand, it is in discrepancy with participants' apparent performance deterioration in functional tests. In the absence of other medical indications for structural assessment, exercise testing seemed sufficiently sensitive to deconditioning at the start of treatment, particularly for muscle strength and cardiorespiratory fitness, to act in place of more elaborate but costly or burdensome clinical examinations. Thereby, patients may benefit from a more straightforward transfer from testing to therapy and therapists can gain insight into patients' deficits and resources beyond structural level (e.g., anxiety to move, health-related behaviors, family support, home training).

Similarly, visceral fat thickness revealed small, variable deviations from a healthy sample [with limited comparability, extracted from the original method reference data; (51)]. Despite the association of abdominal fat and cardio-metabolic risk factors in children and adolescents (68), focusing on area-specific tissue thickness or waist circumference may not be advantageous over broader anthropometric measures such as WtHR or BMI, especially in the absence of standardized procedures and age- and sex-specific norm values. Yet, the latter did also not reveal increased fat mass and reduced fat-free mass previously seen in children undergoing leukemia treatment (69). Thus, measures and metrics of overall body fat and fat-free mass (e.g., bioimpedance analysis) may be needed to retrace expected changes in body composition.

At baseline and the following week, the present sample did not exhibit any hyperglycemia, which adds to the highly variable incidence recorded in the literature [3%–63%; (5, 14]. However, the absence of glycemic deterioration does not necessarily reflect clinical insignificance of testing, especially in the light of ongoing controversies about different clinical definitions and the impact of sample characteristics (5, 14, 70). For example, one third of included patients in the present study were diagnosed with HL, which has been associated with fewer cases of hyperglycemia (70) and in the absence of other common risk factors such as being overweight or obese.

Taken together with a seemingly lower prevalence of hypertriglyceridemia and dyslipidemia than previously reported [e.g., >90% at diagnosis; (55)] metabolic profiles of participants remain highly variable and therefore inconclusive.

### 4.4 Exploration of outcome trajectories

Overall, descriptive changes over time indicate complex and highly individual courses of physical performance, functional capacity, body composition, and metabolic health. However, influencing factors cannot be inferred from the present study, as results may not only reflect consequences of the disease, treatment, or intervention, but also other, for instance, behavioral factors related to reduced levels of physical activity, extensive sedentary or bedrest. Accordingly, results should be interpreted with much caution regarding domains affected, as well as their direction, extent, and time of onset.

Physical performance and functional capacity declined during early therapy phases, with the most pronounced changes occurring between baseline (t0) and week four (t2). By week eight (t3), impairments in hand grip strength and long jump distance largely persisted suggesting a somewhat incomplete recovery after induction therapy. In contrast, diverging courses of SRT peak power and peak heart rate highlight heterogeneous performance trajectories across domains and between subjects. This supports previous notions of reduced muscle strength and functional test performance as early as the end of induction therapy (2, 71, 72) and proposals of timely assessment. Partial recovery was observed between week four and week eight, suggesting early signs of stabilization when transitioning into consolidation therapy, which corresponds to a similar pattern shown in other studies (2, 71, 72). Despite later recovery, a general need for intervention presumably persists, as performance has been shown to remain inferior to healthy norms elsewhere, both throughout and until the end of acute treatment (72, 73), as well as thereafter in survivorship (17, 74).

Sonographic results of quadriceps thickness are in line with previously reported reductions of muscle quantity, proposing a relatively greater loss of skeletal muscle mass during early therapy [<1.5 months; (1)]. On the contrary, the present sample does not exhibit marked increases in visceral fat thickness or anthropometric measures that would imply a shift of body composition as consistently observed in other studies (1). Potentially, such changes are masked by methodological differences, for instance, assessing only segmental and not total body fat, or using measures derived from total body mass, which in turn remain largely unaffected by concurrent sarcopenia and increase of fat mass (1). Moreover, marked increases in body fat have been found to also arise at later stages of therapy, accompanying early recovery of muscle quantity (1), which may not (yet) be observable in this sample.

Regarding metabolic health, missing longitudinal data further limits preliminary analysis. Fasting and postprandial blood glucose levels at baseline and thereafter did not reflect any consistent tendency, neither positive nor pathological (e.g., hyperglycemia). Despite frequently delayed sampling in the present study, fluctuating hypertriglyceridemia [up to Grade 2; (47)] and dyslipidemia continued to be present after baseline in only few patients, supporting the idea that unfavorable metabolic states can rapidly emerge, manifest, and/or fade during induction therapy (55). Thus, a single phenomenon does not seem representative of patients' metabolic health, but may contribute to a bigger picture, considering individual risk factors and critical periods over the course of complex treatment. For instance, episodes of more severe hypertriglyceridemia have been proposed to occur after concurrent steroids and asparaginase, commonly administered during re-induction therapy (75).

### 4.5 Implications for future trials

Firstly, considering the variety of physical and functional performance assessments available and previously used in the pediatric cancer population, the present results put forward the idea oftailored testing. Besides consequently limited comparability between individuals and studies, a toolbox approach may be applicable in both research and therapy practice to allow for risk stratification and timely detection of expected functional decline (34, 35). Also, proxy tests (e.g., 5-T STS) may be a feasible alternative if safety concerns or severe physical limitations are likely to cause missing data (e.g., standing long jump).

Secondly, physical and functional domains affected seemed highly variable in magnitude. Since clinical relevance of single outcomes remains to be established, prioritizing and refining corresponding assessments seem worthwhile. Ideally, this includes test validity and reliability in patients, eliminating duplicates and crosstalk of outcomes, and determining population-specific references.

Thirdly, organizational or hospitalization-related constraints interfering with strict study protocols may advise a universal anchor when defining and reporting the time of assessments. In hematologic malignancies, the day of diagnosis appears to be less feasible than the start of therapy because, treatment for leukemia typically starts immediately, whereas lymphoma patients await staging procedures first. If applicable, prolonged periods for baseline assessment or partial examination over several days may be reasonable to alleviate time pressure for obtaining informed consent, diagnostics, and establishing the patient-therapist relationship.

### 4.6 Limitations

Given the nature of a pilot trial, limitations of this study include the very small sample size, limited implementation fidelity in terms of training adherence, not blinded, delayed and missing assessments, as well as unmeasured confounding factors. For example, overall training volume and intensity as well as intra- and inter-individual differences may be confounded and potentially higher than estimated from therapy logs. Special consideration must be given to recruitment and a potentially underlying selection bias as acceptability of the intervention may be different in patients with less positive general attitudes towards exercise. Furthermore, insufficient randomization, matching and intervention received did not allow for formal exploration of intervention effects.

By including only hematologic malignancies, treatment heterogeneity was already reduced compared to studies that also include solid tumors. Nevertheless, therapy protocols, medication and particularly corticosteroids used, as well as deviations from original treatment protocol varied between participants and potentially affect both patient- and study-level outcomes. In the future, broadening inclusion criteria (e.g., age, time between diagnosis and inclusion) or adopting a multicenter design may improve these shortcomings.

With regards to assessments, consistent blinding of assessors was only achieved in sonography, blood sampling, and anthropometric measures but not in exercise testing due to limited availability of external staff to the strict timeline of follow-ups. Thus, related outcomes may be subject to timing and/or measurement bias. Regarding ultrasound, the use of a single trained operator in the present study minimized inter-operator variability, thereby strengthened internal consistency of the measurements, but also accounted for high proportions of delayed or missing data. Thus, future studies may benefit from including multiple operators that further allow for assessment of inter-rater reliability. Another limitation inherent to sonography is that probe-induced compression, despite liberal gel use, cannot be entirely excluded, particularly in adipose tissue measurements. Also, other imaging techniques such as computed tomography or magnetic resonance imaging may offer more sensitive or accurate structural assessments.

Finally, subjective evaluation based on closed questions may be time-efficient and easier to answer for children and adolescents but limits feedback to certain aspects of interest. If applicable, semi-structured interviews with open options for response or separate parent-based evaluation could add to a more comprehensive understanding of a family's experience during early therapy and concurrent exercise interventions.

## 5 Conclusion

Recruiting newly diagnosed adolescents with hematologic malignancies to a complex exercise–nutrition intervention while undergoing induction therapy was challenging and advocates for adaptation of key aspects in future trials. Intervention dosage and delivery should account for symptom burden, organizational constraints and frequent medical contraindications, for example by integrating low-threshold, adaptable sessions, remote formats, and close coordination with treating teams.

Still, repeated physical assessments and one weekly exercise session with a volume of about 30 min seemed feasible for patients during early intensive treatment. Results suggest that patients' physical functional performance is reduced at baseline and may further decline within the first eight weeks of treatment. Exercise testing appears to be helpful to detect physical deficits, individualize training, and educate patients about deconditioning and health-related behaviors. In contrast, initial status and trajectories of body composition and metabolic health were highly variable and remain inconclusive.

## Data Availability

The original contributions presented in the study are included in the article/Supplementary Material, further inquiries can be directed to the corresponding author/s.
