# Supplementary material for: Feasibility of exercise training to address corticosteroid-induced changes in adolescents aged 10+ with lymphoma or leukemia during the first eight weeks of treatment: a pilot study
Source: Front Pediatr. 2026 Oct 7;14:1819451. doi: 10.3389/fped.2026.1819451 (PMC13683610; doi:10.3389/fped.2026.1819451)
Supplement: Supplementary file 1 [file Supplementaryfile1.docx]

Supplementary Material

# 1 Trial design

Figure 1. Schematic representation of pilot trial.

Legend: The study comprises baseline assessment (t0) and three follow-up tests during intervention (t1-3), as well as one post intervention (t4). The intervention includes a one-time nutrition consultation and exercise program within the first eight weeks of therapy. Participants were allocated to one of two parallel arms using stratified randomization. The multimodal intervention group involves aerobic and resistance exercises at moderate to vigorous intensities. The sham control group involves flexibility, relaxation, and fine motor coordination exercises at low-level intensity. RPE = rating of perceived exertion.

# 2 Methods

## 2.1 Physical performance and functional capacity test procedures

### 2.1.1 Five-times sit-to-stand test

Patients were asked to stand up from a stool and sit back down five times, as quickly as possible and with arms crossed in front of the chest. Movement quality was controlled by full knee and hip extension in standing position, as well as a brief touch-down on the seat when sitting down. Test duration was aggregated for each patient and time point over three consecutive trials (as mean and standard deviation).

### 2.1.2 Hand grip strength

In a standardized sitting position (1), patients were asked to squeeze a handheld dynamometer (adjustable for hand size; Jamar Hydraulic Hand Dynamometer, Performance Health, Downers Grove, Illinois, USA) as much as possible while investigators verbally encouraged performance. Isometric maximum hand grip strength was recorded to nearest 0.5 kilogram and quantified as mean and standard deviation from three alternating trials for each hand.

### 2.1.3 Cycle ergometry

Patients completed a minimally loaded (10 watt), three-minute warm-up on a cycling ergometer, immediately followed by the original steep ramp test protocol (SRT; 2). Cycling load increased by 25 watt every 10 seconds (irrespective of height, gender or age), while participants were asked to pedal at 60-80 revolutions per minute (rpm) for as long as possible. Peak power, peak heart rate, and total test duration were obtained when patients dropped below 50 rpm and could not continue despite assessor’s verbal encouragement.

## 2.2 Example questions from patient/parent questionnaire:

- “The exercise program of the last eight weeks was…”

(1 = “physically very demanding”, 5 = “physically not demanding at all”)

- “I would have preferred the exercise program of the last eight weeks to be conducted online.”

(1 = “I strongly disagree”, 5 = “I strongly agree”)

- “In the last eight weeks, I enjoyed exercising.”

(1 = “I strongly disagree”, 5 = “I strongly agree”)

- “The sport program was an additional burden during early therapy.”

(1 = “I strongly disagree”, 5 = “I strongly agree”)

- “The exercise program affected my mood.”
- “The exercise program affected my self-confidence.”

(0 = “no change”, 1 = “markedly worsened”, 5 = “markedly improved”)

Table 1. Corticosteroid treatment schemes

| **Treatment protocol** | **Corticosteroid prescribed** | **Full dose**  **(cumulative)** | **Administration**  **(Intravenous or oral)** | **Additional information** |
| --- | --- | --- | --- | --- |
| AIEOP-BFM ALL 2017  (n = 3) | a) PRED  b) DEXA | a) 60 mg/m²/day  (1800 mg/m²)  b) 10 mg/m²/day  (70 mg/m²) | a) In one block for 21 days  b) In one block for 7 days | a) Gradual uptake of full dose; followed by up to 9 additional days of tapering.  b) Applicable to patients stratified to early high-risk group. |
| Euronet-PHL-C2 (n = 2) | a) PRED  b) PRED | a) 60 mg/m²/day   (1800 mg/m²)   b) 40 mg/m²/day   (600 mg/m²) | a) In two blocks for 15 days each (13 days in between)  b) In four blocks for 8 days each (13 days in between) | No tapering. |
| ALCL-VBL  (n = 1) | a) PRED  b) DEXA | a) 10 mg  b) 10 mg/day   (50 mg) | a) One intrathecal installation  b) In one block for 5 days | No tapering. |

Legend: Overview of daily and cumulative corticosteroid dosage prescribed in treatment protocol or guideline that was concurrently administered to the duration of the pilot trial (until T4). Individual doses are calculated based on body surface area in m². More information on clinical studies and respective treatment protocols can be derived from [www.gpoh.de/kinderkrebsinfo](https://www.gpoh.de/kinderkrebsinfo/content/health_professionals/clinical_trials/therapy_trials_and_registries/index_eng.html.).

Table 2. Overview of patient-level outcome domains, measures, and metrics.

| **Domain** | **Measure / Test** | **Metric** | **Reference** |
| --- | --- | --- | --- |
| Physical performance  and functional capacity | 5-T STS | Mean duration of 3 consecutive trials [s] | (3, 4) |
|  | Hand grip strength | Mean max. power of 3 trials [kg] | (1, 4) |
|  | SRT cycle ergometry | Peak power [w], peak heartrate [bpm] | (5, 2) |
|  | Finger tapping | Mean tap count of 3 consecutive trials | (6) |
|  | Medicine ball shot | Max. throwing distance [m], best of 2 trials | (7) |
|  | Single leg stance | Number of errors in 60 seconds | (7, 8) |
|  | Standing long jump | Max. jumping distance [m], best of 2 trials | (8) |
| Body composition | US fat | Visceral fat thickness [cm] | (9) |
|  | US muscle | Quadriceps muscle thickness [cm] | (10, 11) |
|  | Body mass index | Ratio of body weight to body height [kg/m²] | (12) |
|  | WtHR | Ratio of waist circumference to body  height [cm/cm] | (13, 12) |
| Metabolic health | Blood glucose | Fasting and postprandial glucose [mg/dl] | (14) |
|  | Insulin | Fasting insulin level [μU/ml] | (14) |
|  | Insulin resistance | HOMA-IR [(μU/ml)/(mg/dl)] | (14, 15) |
|  | Longterm blood glucose | HbA1c level [mmol/mol] | (16) |
|  | Bowel movement | Incidence of unusual bowel movements | - |
|  | Cholesterol | Total, HDL, and LDL cholesterol levels | (17, 18) |
|  | Hyperglycemia | Incidence of hyperglycemia | (19) |
|  | Triglycerides | Triglyceride levels [mg/dl] | (14) |

Legend: Primary and secondary outcomes of each domain are presented in non-shaded and shaded cells, respectively. 5-T STS = Five-times sit-to-stand test. HbA1c = Hemoglobin A1c. HDL = High-density lipoprotein. HOMA-IR = Homeostatic Model Assessment for Insulin Resistance. LDL = Low-density lipoprotein. SRT = Steep ramp test protocol. US = Ultrasound. WtHR = Waist-to-height ratio.

# 3 Results

Table 3. Feasibility outcomes of implementation fidelity and safety.

|  | Overall, n= 6  Median (IQR) | Intervention, n = 2  Min - Max | Control, n = 4  Median (IQR) |
| --- | --- | --- | --- |
| Exercise sessions |  |  |  |
| Sessions completed / offered | 39 / 77 | 6 / 22 | 33 / 55 |
| Sessions completed at target intensity | 89.7% | 100% | 87.9% |
| Sessions completed  per participant | 5.5 (3.5 - 11.0) | 2.0 - 6.5 | 5.5 (4.3 - 9.8) |
| Total volume [minutes]  per participant | 185.0 (102.5 - 325.0) | 50.0 - 185.0 | 185.0 (135.0 - 302.5) |
| Session frequency per week  per participant | 0.7 (0.4 - 1.4) | 0.3 - 0.8 | 0.7 (0.5 - 1.2) |
| Session duration [minutes] | 30.5 (28.1 - 32.8) | 25.0 - 27.0 | 31.3 (30.2 - 34.9) |
| Session intensity [Borg RPE] | 9.8 (8.0 - 13.9) | 10.9 - 13.4 | 8.4 (8.0 - 12.2) |
| Exercise omission |  |  |  |
| Weeks without training  overall | 14 / 48 | 8 / 16 | 6 / 32 |
| Weeks without training  per participant | 2.5 (0.8 - 3.5) | 0.0 - 2.5 | 2.5 (1.3 - 3.0) |
| Incomplete measurements |  |  |  |
| Incidence overall | 20 / 66 | 8 / 22 | 12 / 44 |
| Incidence per test domain  Blood / motoric / sonography | 13 / 6 / 1 | 4 / 3 / 1 | 9 / 3 / 0 |
| Delayed measurements |  |  |  |
| Incidence overall | 47 / 66 | 15 / 22 | 32 / 44 |
| Incidence per test domain  Blood / motoric / sonography | 19 / 12 / 13 | 7 / 5 / 3 | 15 / 7 / 10 |
| Incidence of adverse events  (CTCAE classification^1^) | 0 (-) | 0 (-) | 0 (-) |
| Risk group post-induction | 1 MR, 2 HR, 2 TL III, 1 - | 1 MR, 1 TL III | 2 HR, 2 TL III, 1 - |

Legend: Absolute counts, reasonably rounded ratios, median, and interquartile or group ranges are presented for primary and secondary outcomes in non-shaded and shaded cells, respectively. ^1^ = (20). IQR = Interquartile range. HR = High risk. RPE = rating of perceived exertion using original Borg scale, 6 = minimum value, 20 = maximum value, (21, 22). SR = Standard risk. TL III = Therapy level III.

Table 4. Outcome trajectories of physical performance and functional capacity.

|  | Difference t0 - t2 | | | Difference t0 - t3 | | | Difference t2 - t3 | | |
| --- | --- | --- | --- | --- | --- | --- | --- | --- | --- |
|  | N | ΔM ± SD | ΔZ | N | ΔM ± SD | ΔZ | N | ΔM ± SD | ΔZ |
| 5-T STS  [s] | 5 | 7.1 ± 12.1 ^a^ | 0.6 | 4 | 4.3 ± 5.5 ^a^ | 0.8 | 4 | -3.5 ± 11.2 ^a^ | -0.3 |
| Grip strength D  [kg] | 6 | -4.9 ± 3.4 | -1.5 | 6 | -7.9 ± 6.3 | -1.3 | 6 | -3.0 ± 6.2 | -0.5 |
| Grip strength ND  [kg] | 6 | -4.8 ± 3.2 | -1.5 | 6 | -7.9 ± 5.9 | -1.3 | 6 | -3.0 ± 6.9 | -0.4 |
| SRT peak power  [w] | 4 | -18.0 ± 15.8 | -1.1 | 6 | 2.8 ± 47.2 | 0.1 | 4 | 15.8 ± 48.1 | 0.3 |
| SRT peak HR  [bpm] | 4 | 11.0 ± 18.0 | 0.6 | 6 | 18.5 ± 21.5 | 0.9 | 4 | 3.0 ± 38.1 | 0.1 |
| SLS  [errors] | 5 | 0.6 ± 9.4 | 0.1 | 5 | 1.2 ± 9.9 | 0.1 | 6 | -2.0 ± 7.1 | -0.3 |
| SLJ  [cm] | 3 | -27.8 ± 58.6 | -0.5 | 5 | -23.0 ± 43.8 | -0.5 | 3 | 18.0 ± 15.3 | 1.2 |
| MBS  [m] | 4 | -0.1 ± 1.3 | -0.1 | 5 | 0.1 ± 0.9 | 0.2 | 5 | 0.1 ± 0.6 | 0.2 |
| TAP  [count] | 6 | 3.0 ± 5.4 | 0.6 | 6 | -0.3 ± 7.2 | -0.04 | 6 | -3.3 ± 10.9 | -0.3 |

Legend: Reasonably rounded mean differences between measurement timepoints are presented including standard deviations and z-scores for primary and secondary outcomes in non-shaded and shaded cells, respectively. 5-T STS = Five-times sit-to-stand test. ^a^ = Positive values indicate slower execution. D = Dominant hand. HR = Heart rate. ND = Non-dominant hand. MBS = Medicine ball shot. SLJ = Standing long jump. SLS = Single leg stance. SRT = Steep ramp test protocol. t0 = Baseline. t2 = Follow-up after four weeks of therapy. t3 = Follow-up after eight weeks of therapy. TAP = Finger tapping.

Table 5. Outcome trajectories of body composition and anthropometry.

|  | Difference t0 -t2 | | | Difference t0 - t3 | | | Difference t2 - t3 | | |
| --- | --- | --- | --- | --- | --- | --- | --- | --- | --- |
|  | N | ΔM ± SD | ΔZ | N | ΔM ± SD | ΔZ | N | ΔM ± SD | ΔZ |
| VFT [cm] | 5 | -0.5 ± 0.7 | -0.8 | 5 | -0.2 ± 0.7 | -0.2 | 6 | 0.5 ± 1.1 | 0.5 |
| QMT [cm] | 5 | -0.6 ± 0.7 | -1.0 | 5 | -1.0 ± 1.4 | -0.7 | 5 | -0.2 ± 1.0 | -0.2 |
| BMI | 5 | -0.19 ± 1.27 | -0.15 | 5 | 0.28 ± 2.23 | 0.12 | 6 | 0.32 ± 3.68 | 0.09 |
| WtHR | 6 | -0.02 ± 0.03 | -0.54 | 6 | -0.01 ± 0.03 | -0.17 | 6 | 0.01 ± 0.04 | 0.21 |

Legend: Reasonably rounded mean differences between measurement timepoints are presented including standard deviations and z-scores for primary and secondary outcomes in non-shaded and shaded cells, respectively. QMT = Quadriceps muscle thickness. VFT = Visceral fat thickness. t0 = Baseline. t2 = Follow-up after four weeks of therapy. t3 = Follow-up after eight weeks of therapy. WtHR = Waist-to-heigh ratio.

Table 6. Outcome trajectories of metabolic health.

|  | Difference t0 - t1 | | | Difference t0 - t2 | | | Difference t0 - t3 | | |
| --- | --- | --- | --- | --- | --- | --- | --- | --- | --- |
|  | N | ΔM ± SD | ΔZ | N | ΔM ± SD | ΔZ | N | ΔM ± SD | ΔZ |
| PP BG [mg/dl] | 4 | -12.5 ± 21.0 | -0.5 | 4 | -12.2 ± 22.9 | -0.6 | 5 | 0.0 ± 14.8 | -0.5 |
| F BG [mg/dl] | 6 | -2.0 ± 11.6 | -0.2 | 6 | -10.0 ± 34.4 | -0.3 | 6 | -7.2 ± 19.4 | -0.4 |
| F insulin [μU/ml] | 2 | - | - | 1 | - | - | 1 | - | - |
| HOMA-IR [(μU/ml)/(mg/dl)] | 2 | - | - | 1 | - | - | 1 | - | - |
| TG [mg/dl] | - | - | - | 5 | -89.2 ± 115.4 | -0.8 | 5 | 9.5 ± 104.2 | 0.1 |
| TCH [mg/dl] | - | - | - | 4 | 27.3 ± 15.9 | 1.7 | 5 | 54.2 ± 68.7 | 0.8 |
| HDL-C [mg/dl] | - | - | - | 3 | 7.5 ± 32.2 | 0.2 | 5 | 14.6 ± 11.3 | 1.3 |
| LDL-C [mg/dl] | - | - | - | 3 | 2.5 ± 25.9 | 0.1 | 5 | 35.0 ± 56.1 | 0.6 |

Legend: Reasonably rounded mean differences between measurement timepoints are presented including standard deviations and z-scores for primary and secondary outcomes in non-shaded and shaded cells, respectively. BG = Blood glucose. F = Fasting. HDL-C = High-density cholesterol. HOMA-IR = Homeostatic Model Assessment for Insulin Resistance. LDL-C = Low-density cholesterol. PP = Postprandial. t0 = Baseline. t1 = Follow-up after one week of therapy. t2 = Follow-up after four weeks of therapy. t3 = Follow-up after eight weeks of therapy. TCH = Total cholesterol. TG = Triglycerides.

# 4 Intervention material

# Supplementary figure 1. Exercise for intervention group poster

Legend: Poster used for exercise sessions with participants allocated to the intervention group. Depicted are warm-up routine, circuit training involving one of five pre-defined exercises per category, and options for cool-down or free play in the end.

Supplementary figure 2. Sham-exercise for control group poster

Legend: Poster used for exercise sessions with participants allocated to the sham-exercise control group. Depicted are different low-intensity or relaxation exercises or games, of which up to five were suggested by therapists or selected by participants per session (with the first and last represented warm-up and cool-down, respectively).

Supplementary figure 3

Legend: Semi-structured interview guideline for nutrition counselling to determine knowledge about dietary status, foot intake, and experience of gastrointestinal treatment-related symptoms. Based on this information, participants received advice regarding food hygiene, storage, cooking, preparation, and services in the clinical setting as well as nutritional goal-setting.

Literature Cited

1. McQuiddy VA, Scheerer CR, Lavalley R, McGrath T, Lin L. Normative Values for Grip and Pinch Strength for 6- to 19-Year-Olds. Arch Phys Med Rehabil 2015; 96(9):1627–33.

2. Braam KI, van Dulmen-Den Broeder E, Veening MA, Merks JH, van den Heuvel-Eibrink MM, Kaspers GJ et al. Application of the steep ramp test for aerobic fitness testing in children with cancer. Eur J Phys Rehabil Med 2015; 51(5):547–55.

3. Bohannon RW, Bubela DJ, Magasi SR, Wang Y-C, Gershon RC. Sit-to-stand test: Performance and determinants across the age-span. Isokinet Exerc Sci 2010; 18(4):235–40.

4. Götte M, Kesting SV, Winter CC, Rosenbaum D, Boos J. Motor performance in children and adolescents with cancer at the end of acute treatment phase. Eur J Pediatr 2015; 174(6):791–9.

5. Bongers BC, Vries SI de, Obeid J, van Buuren S, Helders PJM, Takken T. The Steep Ramp Test in Dutch white children and adolescents: age- and sex-related normative values. Phys Ther 2013; 93(11):1530–9.

6. Skogan AH, Oerbeck B, Christiansen C, Lande HL, Egeland J. Updated developmental norms for fine motor functions as measured by finger tapping speed and the Grooved Pegboard Test. Dev Neuropsychol 2018; 43(7):551–65.

7. Götte M, Kesting S, Albrecht C, Worth A, Bös K, Boos J. MOON-test - determination of motor performance in the pediatric oncology. Klin Padiatr 2013; 225(3):133–7.

8. Tomkinson GR, Carver KD, Atkinson F, Daniell ND, Lewis LK, Fitzgerald JS et al. European normative values for physical fitness in children and adolescents aged 9-17 years: results from 2 779 165 Eurofit performances representing 30 countries. Br J Sports Med 2018; 52(22):1445–14563.

9. Jung JH, Jung MK, Kim KE, Kwon AR, Chae HW, Yoon CS et al. Ultrasound measurement of pediatric visceral fat thickness: correlations with metabolic and liver profiles. Ann Pediatr Endocrinol Metab 2016; 21(2):75–80.

10. Högelin ER, Thulin K, Walden F von, Fornander L, Michno P, Alkner B. Reliability and Validity of an Ultrasound-Based Protocol for Measurement of Quadriceps Muscle Thickness in Children. Front Physiol 2022; 13:830216.

11. Lori S, Lolli F, Molesti E, Bastianelli M, Gabbanini S, Saia V et al. Muscle-ultrasound evaluation in healthy pediatric subjects: Age-related normative data. Muscle Nerve 2018; 58(2):245–50.

12. Rosario AS, Kurth B-M, Stolzenberg H, Ellert U, Neuhauser H. Body mass index percentiles for children and adolescents in Germany based on a nationally representative sample (KiGGS 2003-2006). Eur J Clin Nutr 2010; 64(4):341–9.

13. Eslami M, Pourghazi F, Khazdouz M, Tian J, Pourrostami K, Esmaeili-Abdar Z et al. Optimal cut-off value of waist circumference-to-height ratio to predict central obesity in children and adolescents: A systematic review and meta-analysis of diagnostic studies. Front Nutr 2022; 9:985319.

14. Hammel MC, Stein R, Kratzsch J, Vogel M, Eckert AJ, Triatin RD et al. Fasting indices of glucose-insulin-metabolism across life span and prediction of glycemic deterioration in children with obesity from new diagnostic cut-offs. Lancet Reg Health Eur 2023; 30:100652.

15. Matthews DR, Hosker JP, Rudenski AS, Naylor BA, Treacher DF, Turner RC. Homeostasis model assessment: insulin resistance and beta-cell function from fasting plasma glucose and insulin concentrations in man. Diabetologia 1985; 28(7):412–9.

16. Hovestadt I, Kiess W, Lewien C, Willenberg A, Poulain T, Meigen C et al. HbA1c percentiles and the association between BMI, age, gender, puberty, and HbA1c levels in healthy German children and adolescents. Pediatric Diabetes 2022; 23(2):194–202.

17. Expert panel on integrated guidelines for cardiovascular health and risk reduction in children and adolescents: summary report. Expert panel on integrated guidelines for cardiovascular health and risk reduction in children and adolescents: summary report. Pediatrics 2011; 128 Suppl 5(Suppl 5):S213-56.

18. Schienkiewitz A, Truthmann J, Ernert A, Wiegand S, Schwab KO, Scheidt-Nave C. Age, maturation and serum lipid parameters: findings from the German Health Survey for Children and Adolescents. BMC Public Health 2019; 19(1):1627.

19. Grimes A, Mohamed A, Sopfe J, Hill R, Lynch J. Hyperglycemia During Childhood Cancer Therapy: Incidence, Implications, and Impact on Outcomes. J Natl Cancer Inst Monogr 2019; 2019(54):132–8.

20. National Cancer Institute. Common Terminology Criteria for Adverse Events (CTCAE) v6.0 (MedDRA 28.0); 2025. Available from: URL: https://dctd.cancer.gov/research/ctep-trials/for-sites/adverse-events/ctcae-v6.pdf.

21. Borg G. Perceived exertion as an indicator of somatic stress. Scand J Rehabil Med 1970; 2(2):92–8.

22. Borg GA. Psychophysical bases of perceived exertion. Med Sci Sports Exerc 1982; 14(5):377–81.
